# I’ve just seen a face: further search for face pareidolia in chimpanzees (*Pan troglodytes*)

**DOI:** 10.3389/fpsyg.2024.1508867

**Published:** 2025-01-28

**Authors:** Masaki Tomonaga

**Affiliations:** School of Psychological Sciences, University of Human Environments, Matsuyama, Ehime, Japan

**Keywords:** face perception, chimpanzees, pareidolia, top-down control, oddity discrimination

## Abstract

**Introduction:**

Seeing faces in random patterns, such as in clouds, is known as pareidolia. Two possible mechanisms can cause pareidolia: a bottom-up mechanism that automatically detects inverted triangle or top-heavy patterns, and a top-down mechanism that actively seeks out faces. Pareidolia has been reported in nonhuman animals as well. In chimpanzees, it has been suggested that the bottom-up mechanism is involved in their pareidolic perception, but the extent of the contribution of the top-down mechanism remains unclear. This study investigated the role of topdown control in face detection in chimpanzees.

**Methods:**

After being trained on an oddity task in which they had to select a noise pattern where a face (either human or chimpanzee) or a letter (Kanji characters) was superimposed among three patterns, they were tested with noise patterns that did not contain any target stimuli.

**Results:**

When the average images of the patterns selected by the chimpanzees in these test trials were analyzed and compared with those that were not selected (i.e., difference images), a clear non-random structure was found in the difference images. In contrast, such structures were not evident in the difference images obtained by assuming that one of the three patterns was randomly selected.

**Discussion:**

These results suggest that chimpanzees may have been attempting to find “faces” or “letters”in random patterns possibly through some form of top-down processing.

## Introduction

Humans often find meaningful patterns in various objects and textures, a phenomenon known as pareidolia (Gardner, 1985; see also Flessert, 2022; Zhou and Meng, 2020 for review). There are different types of pareidolic perception, with face pareidolia being particularly well-studied from psychological, neuropsychological, and neuroscientific perspectives (e.g., Liu et al., 2014; Rolf et al., 2020; Takahashi and Watanabe, 2015; Wardle et al., 2020; Yokoi et al., 2014). Research suggests that two main mechanisms are involved in face pareidolia: bottom-up and top-down. The bottom-up mechanism automatically processes a distinctive facial structure, typically an inverted triangular pattern with two horizontally aligned shapes at the top and one shape below, known as a “top-heavy” pattern (e.g., Kato and Mugitani, 2015; Morton and Johnson, 1991; Takahashi and Watanabe, 2015). In contrast, the top-down mechanism, often triggered by verbal instructions, actively searches for facial features even when the top-heavy pattern is not clearly present (Hansen et al., 2010; Liu et al., 2014; Pavlova et al., 2020; Rieth et al., 2011; Romagnano et al., 2024; Zhou and Meng, 2020).

Takahashi and Watanabe (2015) examined the relationship between these two mechanisms. They conducted an experiment in which participants judged whether briefly presented figures were faces, inverted triangles, or noise patterns. There were two conditions: one for detecting faces and the other for detecting inverted triangles, with a top-heavy pattern appearing in both. In each condition, participants’ expectancies were controlled in a bottom-up manner by presenting either face or inverted triangle figures. Simultaneously, verbal instructions to “look for faces (or triangles)” were given to encourage top-down control. As a result, despite detecting the same top-heavy pattern, the detection rate was higher in the face condition compared to the triangle condition. These results clearly indicate that top-down processing plays a significant role in face pareidolia.

Several studies have visualized what participants perceived during face pareidolia under top-down control using the classification image or reverse correlation method (Gosselin and Schyns, 2003; Hansen et al., 2010; Liu et al., 2014; Rieth et al., 2011; see Murray, 2011 for review). For example, Hansen et al. (2010) conducted an experiment where participants rated whether fractal noise patterns contained faces. Comparing the classification images, derived from the difference between patterns rated as containing a face and those rated as not, to an average face embedded in noise, Fourier analysis revealed similar amplitude spectra. In a subsequent experiment, EEGs were recorded during the behavioral task. When strong negative amplitudes around 170 ms (N170) were observed, classification images with face-like structures appeared in the theta to gamma frequency bands. Similar findings were reported by Rieth et al. (2011) and Liu et al. (2014) using two-dimensional Gaussian noise patterns.

There have been a few studies on face pareidolia in non-human primates (Flessert et al., 2023; Kuwahata et al., 2003, 2004; Myowa-Yamakoshi and Tomonaga, 2001; Taubert et al., 2017, 2022; Tomonaga, 2007). Notably, the perception of top-heavy patterns has been observed in infant chimpanzees, gibbons, and macaques (Kuwahata et al., 2003, 2004; Myowa-Yamakoshi and Tomonaga, 2001). Free-viewing tasks using photographs of objects that resemble faces (face-like objects) have demonstrated face pareidolia, with brain regions responsive to actual face stimuli also reacting to face-like stimuli (Taubert et al., 2017). However, in discrimination tasks, face discrimination using real face stimuli does not generalize to face-like stimuli in capuchin monkeys and rhesus macaques (Flessert et al., 2023).

Chimpanzees have shown efficient search for upright faces compared to inverted faces in visual search tasks (Tomonaga, 1999, 2007). This upright face superiority was also evident when extremely schematic faces were used (Tomonaga, 2007). Furthermore, Tomonaga and Kawakami (2022) tested the chimpanzees on face pareidolia using visual search tasks with photographs of face-like objects. The chimpanzees successfully detected face-like objects among various non-face objects, and their performance declined when the stimuli were horizontally misaligned, disrupting the facial configuration (cf. Taubert et al., 2012; Young et al., 1987). This decline was not observed when photographs of fruits were used as target stimuli. These findings suggest that chimpanzees may process stimuli containing face-like structures, such as top-heavy configurations, as “faces” in a bottom-up manner.

However, in matching-to-sample tasks, the chimpanzees failed to select face-like stimuli when the sample was a real face, and vice versa (Tomonaga and Kawakami, 2022). Matching-to-sample tasks require the subject to select either an identical stimulus or one from the same category as the sample, implying that this behavior involves some degree of top-down control, as the sample explicitly indicates the category to be chosen. The failure of the chimpanzees to match real faces with face-like stimuli in these tasks suggests that top-down processing may not play a significant role in their experience of face pareidolia.

In the present study, we further investigated pareidolia in chimpanzees. Previous humans studies explicitly instructed participants to “find the face,” facilitating top-down processing of face-like objects (Hansen et al., 2010; Liu et al., 2014; Rieth et al., 2011; Takahashi and Watanabe, 2015). While effective for humans, this method is inapplicable to human infants or non-human animals who do not understand such verbal instructions. Therefore, this study aimed to “simulate” top-down control by increasing attentional focus or “expectancy” toward a specific stimulus category through repeated exposure, based on the effects of repetition or sequential priming. In humans, repeated presentation of the same stimulus or category has been shown to enhance task performance (Cameron et al., 2012; Found and Müller, 1996; Hayashi and Kumada, 1999; Logan, 1990; McCarley and He, 2001; Scarborough et al., 1977; Treisman, 1988). Similarly, sequential priming effects in visual search tasks have been reported in non-human animals, including chimpanzees (Tomonaga, 1993), pigeons (Blough, 1989, 1991; Blough and Lacourse, 1994; Bond and Riley, 1991), and blue jays (Bond and Kamil, 1999; Pietrewicz and Kamil, 1979). Such facilitative effects induced by sequential priming may be closely related to the “search image” proposed by von Uexküll (Bond and Riley, 1991; Pietrewicz and Kamil, 1979; von Uexküll, 1934; Tønnessen, 2018). In other words, we employed a kind of top-down control through the formation of search images in the present study.

We employed an oddity discrimination task, where chimpanzees selected the target noise pattern containing an embedded face or letter from among three noise patterns. Human studies often use a yes-no task, asking participants to report whether a face or letter is embedded within a single pattern (e.g., Hansen et al., 2010; Liu et al., 2014; Rieth et al., 2011). However, we chose the oddity discrimination task (e.g., Komaki, 1991; Nissen and McCulloch, 1937; Tomonaga and Imura, 2010), a kind of forced-choice tasks, as it is more suitable for the chimpanzees in the present study, who were well-trained in this task (cf. Tomonaga, 2001, 2010).

The three stimulus categories, human faces, chimpanzee faces, and letters, were used. Rather than presenting these categories randomly within a session, the same category was consistently repeated throughout the preliminary training and the subsequent 25 test sessions. This repetition aimed to establish an “attentional control setting” or learned expectancy akin to top-down control.

The test sessions included baseline trials, where a target stimulus was presented, and test trials, where all stimuli consisted of noise patterns without any target stimuli. If the chimpanzees anticipated a specific stimulus category due to sequential priming, they would likely search for that category even in the test trials. To visualize what the chimpanzees were searching for in the noise patterns, we created classification images by comparing the average images of the selected patterns with those not selected. While standard classification image experiments require a large number of trials, we analyzed data from 300 test trials per category, comparable to the study by Liu et al. (2014), in which 480 patterns were used. If the chimpanzees were indeed searching for the target stimulus within the noise patterns, non-random structures should appear in the difference images. Conversely, if they were guessing, no such structures would appear.

## Methods

### Participants

In the present experiment, five chimpanzees (*Pan troglodytes*) participated: Ai (female, 35 years old at the beginning of the present experiment, Great Ape Information Network (GAIN)^1^ ID#0434), Ayumu (male,11 years old, #0608), Chloe (female, 30 years old, #0441), Cleo (female, 11 years old, #0609), and Pendesa (female, 34 years old, #0095). They had participated in various computer-controlled perceptual and cognitive experiments, such as visual search, oddity discrimination tasks, and face pareidolia (Dahl et al., 2013; Matsuzawa et al., 2006; Tomonaga, 2001, 2010; Tomonaga and Imura, 2010, 2015, 2023b; Tomonaga and Kawakami, 2022; Tomonaga et al., 2003). They lived in a social group of 14 individuals within an indoor area and environmentally enriched outdoor compounds (770 m^2^) at the Primate Research Institute, Kyoto University (Matsuzawa, 2006). In this experiment, no food or water deprivation was employed.

### Ethics statement

For the care and use of the chimpanzees, we followed the 3rd edition of the institute’s *Guide for the Care and Use of Laboratory Primates*. Experimental designs of the present study with chimpanzees were approved by the Animal Welfare and Animal Care Committee of the institute (2011–078, 2012–041). All procedures also adhered to the *Guideline of the Animal Experimentation* of the Japanese Society of Animal Psychology, *Guideline for the Care and Experimental Use of Captive Primates* of the Primate Society of Japan, *Code of Ethics and Conduct* of the Japanese Psychological Association, and Japanese *Act on Welfare and Management of Animals*.

### Experimental setting

Experimental sessions were conducted in a booth (1.8 × 2.15 × 1.75 m) located in a laboratory adjacent to the chimpanzee facility. The chimpanzees accessed the booth via an overhead pathway connecting the facility to the booth. Two 17-inch LCD monitors (I-O Data LCD-AD172F2-T, 1280 × 1,024 pixels, pixel size: 0.264 mm x 0.264 mm) with touch panels were installed on the booth wall. The viewing distance was approximately 40 cm. Food rewards (small pieces of apple) were provided through food dispensers (Biomedica BUF-310) positioned outside the booth. Computers controlled all equipment and experimental events.

### Stimuli

**Figure 1:**
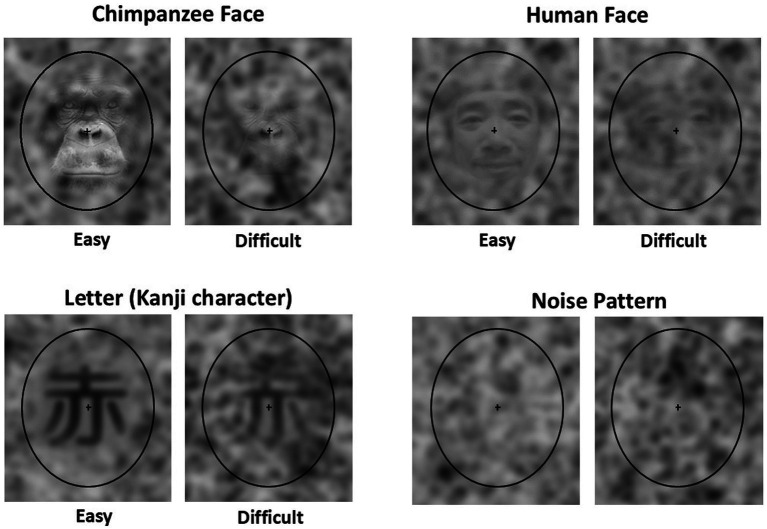
Examples of stimuli used in the present experiment. Easy: The target stimulus in the Easy baseline trial, Difficult: The target stimulus in the Difficult baseline trial, Noise pattern: The distractors used in the baseline trials and the stimuli used in the test trials. Note that the human face stimuli shown here differ from the actual stimuli used due to portrait rights considerations.

In the present experiment, three categories of stimuli were prepared (Figure 1): chimpanzee faces, human faces, and letters. Photographs of chimpanzees living at the Kyoto University Kumamoto Sanctuary were used for the chimpanzee faces. The human faces consisted of photographs of Asian male individuals used in the previous studies (Liu et al., 2014; Rieth et al., 2011). The letter stimuli were Kanji characters. Twenty different stimuli were prepared for each category. All stimuli were converted to grayscale, resized to 200 × 220 pixels, and superimposed onto the center of 300 × 350 noise patterns (see below) using Adobe Photoshop®. Two types of stimulus sets were created by adjusting the opacity of the layers: one easily recognizable and one difficult to recognize. For each stimulus, 100 variations were created by superimposing them on different noise patterns. Each stimulus was enclosed within a black elliptical frame of 238 × 294 pixels, with a 12 × 12 pixel cross added at the center.

The noise patterns used were two-dimensional Gaussian noise patterns, generated in the same manner as described by Liu et al. (2014) and Rieth et al. (2011). Approximately 30,000 noise patterns were created.

### Procedure

**Figure 2:**
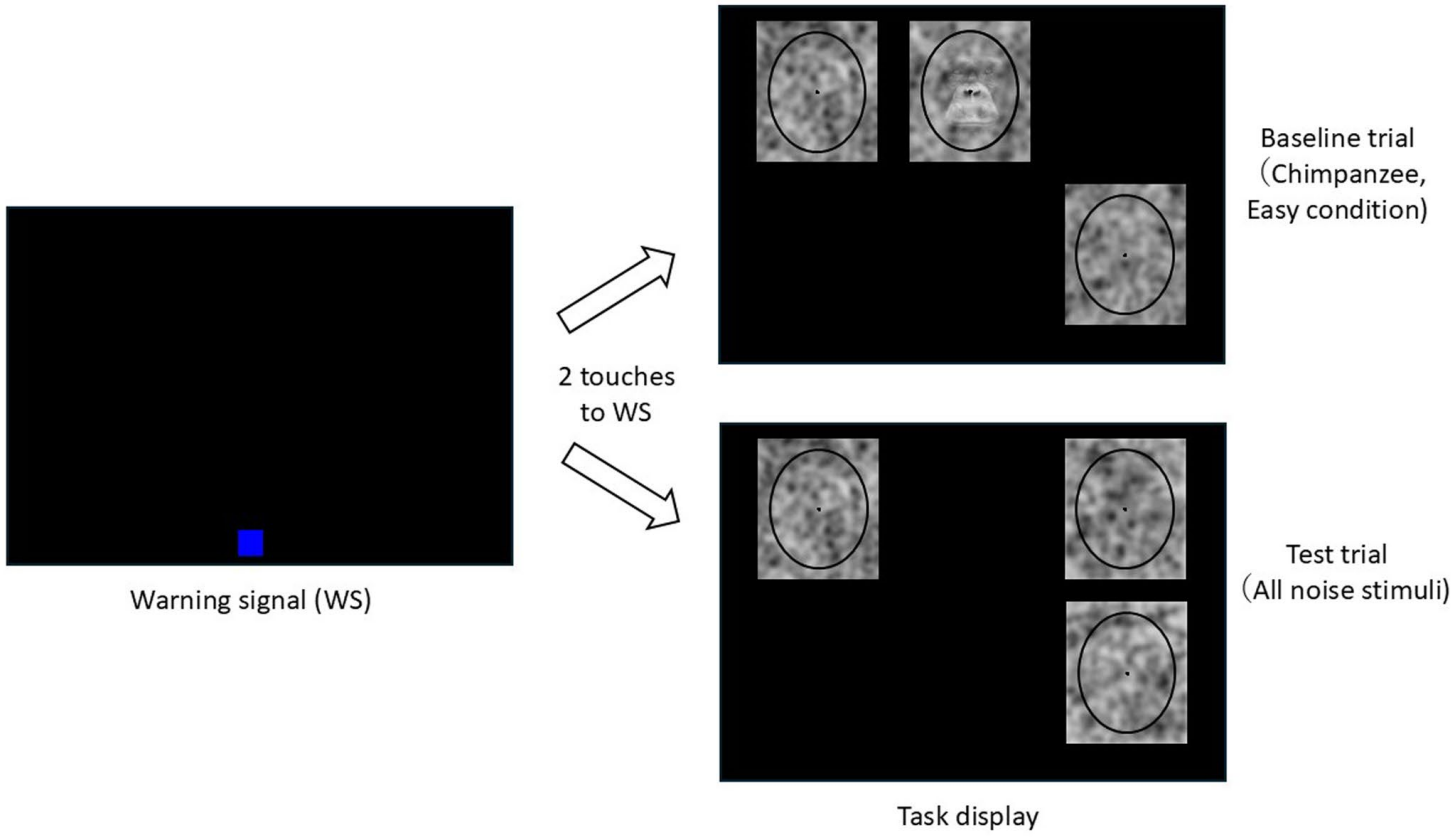
Schematic diagram of the oddity discrimination tasks.

In this experiment, an oddity discrimination task was employed (Figure 2). The chimpanzees were required to detect and touch the target stimulus from among three stimuli, where the target stimulus had a face or letter superimposed on it. The remaining two stimuli, serving as distractors, did not contain any faces or letters and differed from each other.

Figure 2 illustrates the flow of baseline and test trials. Each trial began with the presentation of a blue warning signal (WS, 100 × 100 pixels) at a random position on the lower part of the black monitor screen. If the chimpanzee touched the WS twice, it disappeared, and three stimuli were then presented on the screen at random positions within a predetermined 3 × 2 grid. If the chimpanzee selected the target stimulus in the baseline trial, a chime sounded, and a food reward was given. If an incorrect stimulus was selected, only a buzzer sounded. A correction procedure was employed, where, following an incorrect trial, only the target stimulus from the previous trial was presented.

In the test trials, all three stimuli were noise patterns. Each noise pattern was unique across all trials for each chimpanzee. Regardless of which stimulus was selected in the test trials, the chimpanzee received a food reward 50% of the time. This procedure was introduced to prevent inappropriate incidental learning and the formation of position biases.

The experiment was conducted sequentially, starting with chimpanzee faces, followed by human faces, and then letters. Preliminary training for each stimulus set was first conducted using only easy stimuli, followed by training with difficult stimuli. Each session consisted of 48 trials. After the completion of this preliminary training, the experiment proceeded to the test sessions. Each test session consisted of 48 trials: 36 baseline trials (24 difficult trials and 12 easy trials) and 12 test trials. These trials were presented in a random order. The positions of the stimuli and the correct positions in the baseline trials were randomized in each trial. A total of 25 test sessions were conducted for each stimulus category. Therefore, the chimpanzees underwent 300 trials for each stimulus category.

### Data analysis

**Figure 3:**
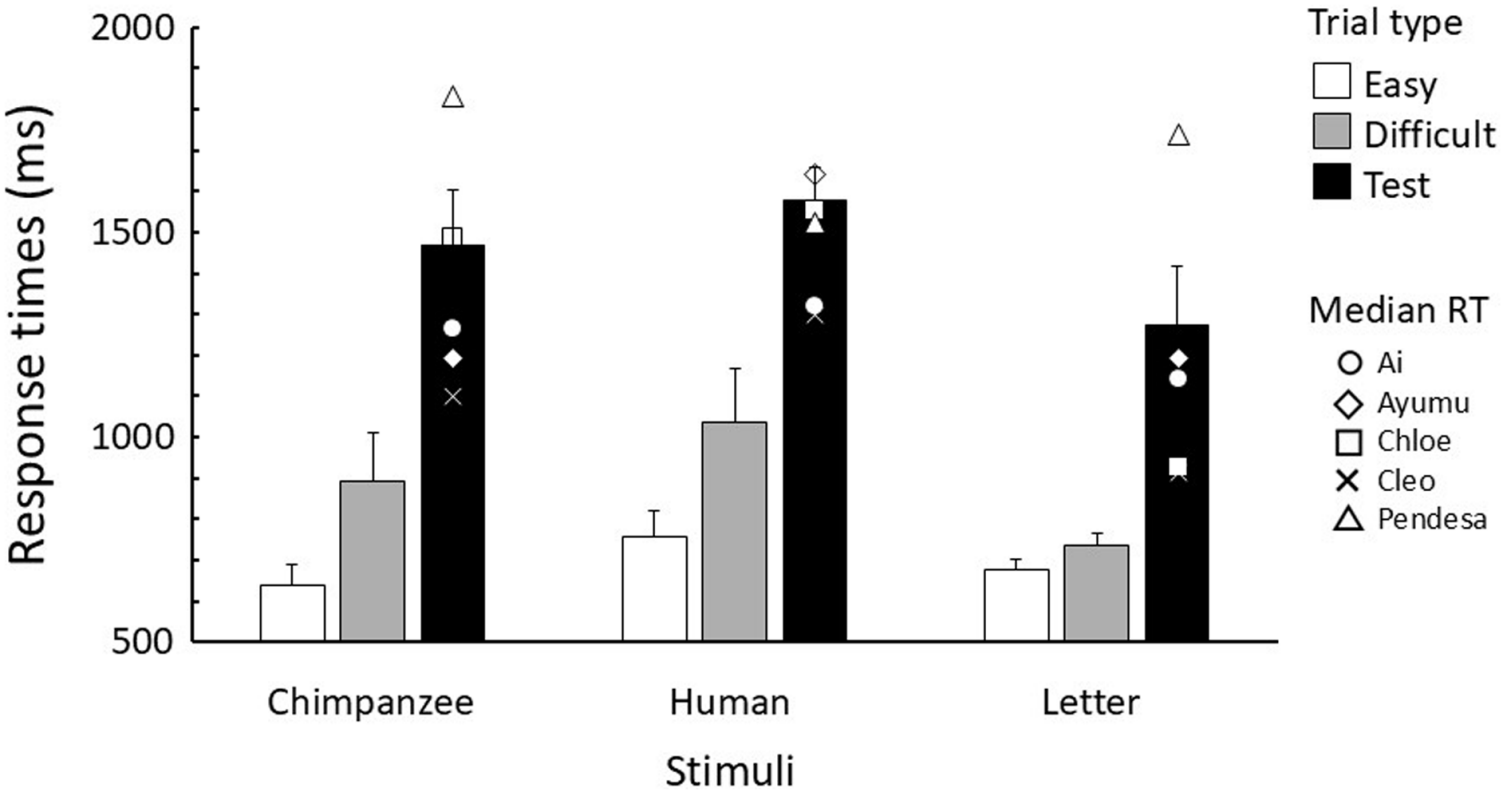
Mean response time for each trial type in the test sessions. Error bars indicate standard error. The symbols in the test trials represent the median response time for each chimpanzee.

The response times in the baseline trials of the test sessions were logarithmically transformed and analyzed using a generalized linear mixed model (GLMM). Data from correction trials were excluded from the analysis, while data from both correct and incorrect trials were included. These analyses were performed using the *lmerTest* package (Kuznetsova et al., 2017) in R version 4.2.0 (R Core Team, 2022). Since the error rates were very low (4.0% averaged across chimpanzees and stimuli), no statistical analysis was conducted on the errors (Figure 3).

**Figure 4:**
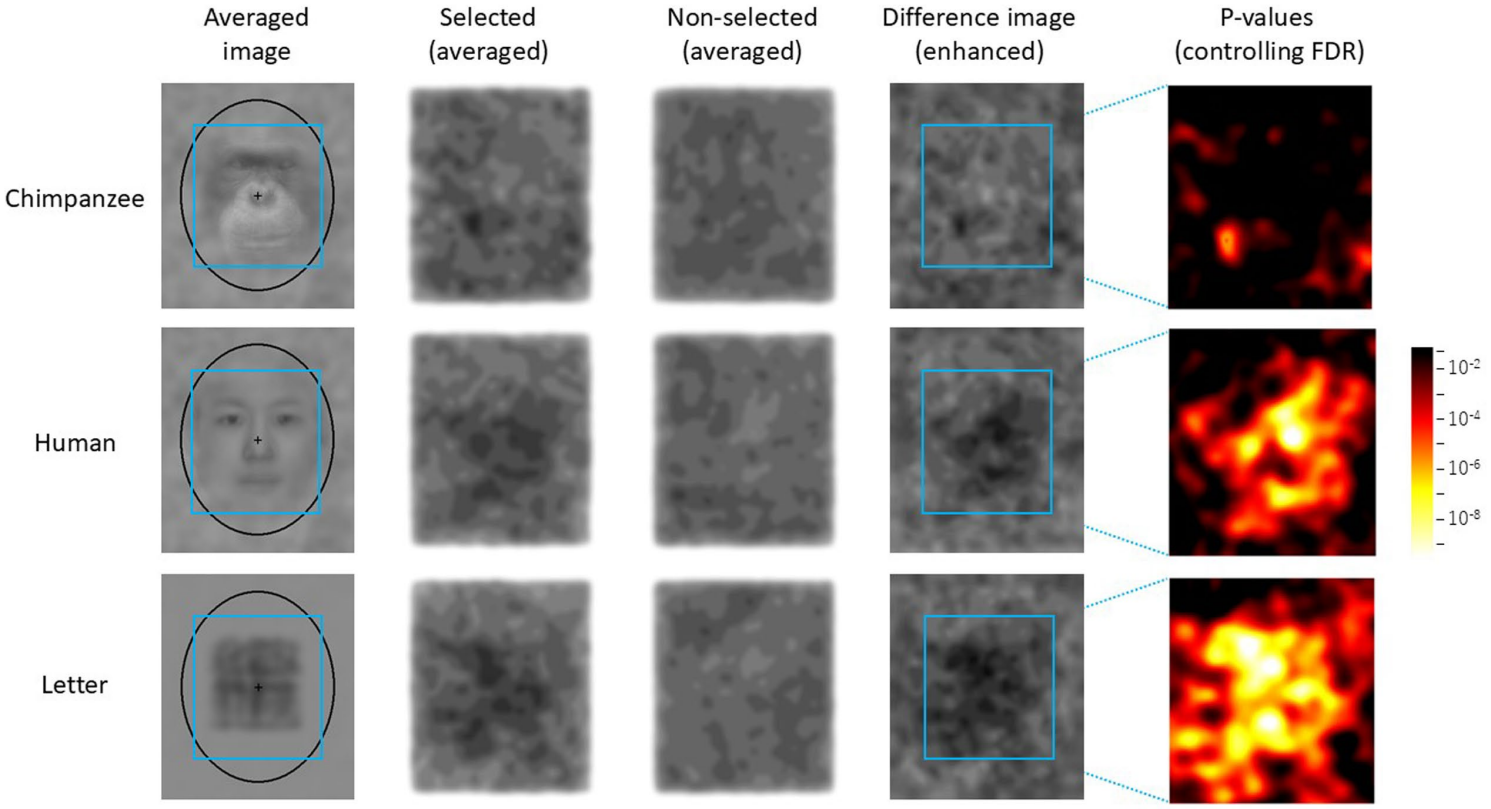
Difference image analysis based on data from all chimpanzees for each stimulus category. The light blue frames indicate the AOIs (areas of interest). The leftmost images show the average image for each stimulus category in the Easy trials. Selected: enhanced average image of the noise patterns actually selected by the chimpanzees, Unselected: the enhanced average image of the stimuli that were not selected, Difference Image: the enhanced difference image obtained by subtracting “Unselected” from “Selected.” Heatmap: FDR-controlled *p*-values based on the results of the GLMM at each point.

The results of the test trials were analyzed as follows. First, the luminance (RGB values) of each pixel of each noise pattern selected by each chimpanzee was recorded. Similarly, for the two stimuli that were not selected, the average luminance of the corresponding coordinates was recorded. As a result, data from 300 trials were collected for each stimulus category. Next, using this dataset, average images of the selected and unselected noise patterns were created for each stimulus category (see Figure 4). When creating these images, the luminance level was enhanced using the following equation:


$$E\left(i,j\right)=\left(L\left(i,j\right)-M\right)\times 20+128\text{,}$$

where E(i, j) is the enhanced luminance at the coordinate (i, j) in the image, L(i, j) is the original luminance, and M is the average luminance of the entire image. Additionally, the difference in luminance between the two images was calculated for each coordinate, and a difference image was created using the above equation, where L(i, j) represented the luminance difference between the two images.

For the statistical analysis, a 200 × 220 area centered on the noise patterns presented in each trial was designated as the area of interest (AOI). A GLMM was conducted separately for each coordinate within the AOI. The fixed factor was the stimulus type (selected versus unselected), and the random factors were the participants (*N* = 5) and trials (*N* = 300, nested within participants). Based on the *p*-values of the parameter estimates for the stimulus type obtained for each coordinate, a heatmap was created. The *p*-values were corrected by controlling the false discovery rate (FDR), which was set at 0.05.

For comparison, a random selection simulation was also performed. In this simulation, one of the three stimuli presented in each trial was randomly selected by the computer, and the same analysis as above was applied to this dataset to create difference images and heatmaps.

Due to the small number of participants (*N* = 5), additional GLMMs were conducted for each individual chimpanzee. In these analyses, the only random factor was the trials.

## Results

### Preliminary training

The mean number of preliminary training sessions, averaged across chimpanzees and stimulus categories, was 2.7 sessions (range: 1–7) for easy stimuli and 4.0 sessions (range: 1–11) for difficult stimuli, respectively. Since the chimpanzees had prior experience with visual search and oddity tasks, they performed well from the start of the preliminary training. The overall error rate during the preliminary training was 10.4% (SEM = 3.3%) for easy sessions and 15.7% (SEM = 4.5%) for difficult sessions.

### Test sessions

In the test sessions, the error rate in the baseline trials was 0.3% (SEM = 0.2%) for easy trials and 4.4% (SEM = 2.1%) for difficult trials in the chimpanzee face condition. The error rate for human faces was 0.9% (SEM = 0.4%) for easy trials and 11.8% (SEM = 8.1%) for difficult trials. For Kanji characters, the error rate was 2.1% (SEM = 1.3%) for easy trials and 4.7% (SEM = 1.8%) for difficult trials.

Figure 3 shows the response times in baseline and test trials. The GLMM results revealed that parameter estimates for the stimulus type, trial type, and their interaction were all significant (Table 1). In each stimulus type, response times (RTs) were longer for difficult trials than for easy trials, and longer in test trials compared to the baseline trials. In comparing stimulus types, it was observed that in both difficult and test trials, response times were longest for human stimuli, followed by chimpanzee stimuli, with letter stimuli being the fastest.

**Table 1:** Summary of the generalized linear mixed model analysis for response times in the test sessions.

Stimulus type	Trial type	Estimate	95%CI	
Human	Easy vs Diff	-0.288	-0.309	-0.268
	Easy vs Test	0.423	0.402	0.443
	Diff vs Test	0.711	0.687	0.735
Chimpanzee	Easy vs Diff	-0.306	-0.327	-0.285
	Easy vs Test	0.490	0.469	0.511
	Diff vs Test	0.797	0.772	0.821
Letter	Easy vs Diff	-0.074	-0.095	-0.054
	Easy vs Test	0.508	0.487	0.528
	Diff vs Test	0.582	0.558	0.606

Trial type	Stimulus type	Estimate	95%CI	
Easy	Chimp vs Human	0.170	0.146	0.194
	Chimp vs Letter	0.065	0.041	0.089
	Human vs Letter	-0.105	-0.129	-0.081
Difficult	Chimp vs Human	0.152	0.135	0.169
	Chimp vs Letter	-0.167	-0.184	-0.150
	Human vs Letter	-0.319	-0.336	-0.302
Test	Chimp vs Human	0.085	0.060	0.109
	Chimp vs Letter	-0.150	-0.174	-0.125
	Human vs Letter	-0.234	-0.258	-0.210

Diff: Difficult task, Chimp: Chimpanzee, CI: Confidence interval.

### Difference image analysis

**Figure 5:**
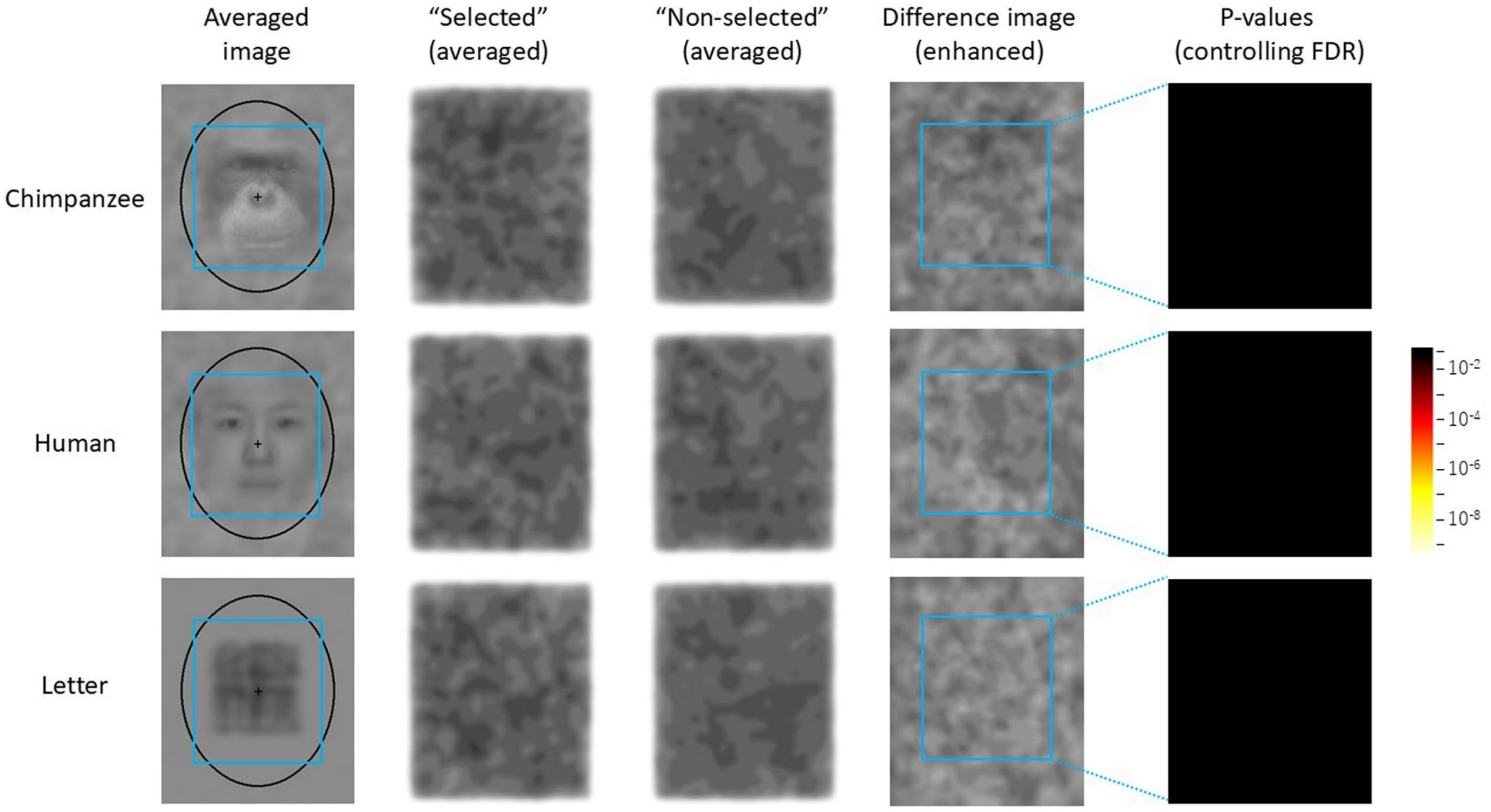
Difference image analysis based on random simulations for each stimulus category.

Figure 4 shows the average noise patterns selected by the chimpanzees during the test trials, the averaged unselected patterns, and the difference images for each stimulus condition. The leftmost column shows the average image for each stimulus condition. The light-blue square frame in the images indicates the AOI. The rightmost heatmap displays the *p*-values for the difference images controlled by FDR. Figure 5 shows the results of the random selection simulation. Figure 4 shows clearly significant regions in the difference image for each stimulus condition, while the difference images obtained from the random selection simulation (Figure 5) did not show any significant regions like those based on the chimpanzee’s actual selections.

From Figure 4, it can be observed that the brightness within the AOI of the difference images (i.e., the difference in brightness between the selected and non-selected images) varied depending on the stimulus type. A GLMM analysis based on the average brightness differences at each point within the AOI revealed that the difference was smallest for chimpanzee stimuli (M = −1.63, SE = 0.71), followed by human stimuli (M = −2.43, SE = 0.35), with letter stimuli (M = −3.03, SE = 0.58) showing the largest average brightness difference (chimpanzee vs. human: *β* = −0.795, [−0.806, −0.784]; chimpanzee vs. letter: *β* = −1.400, [−1.412, −1.389]; human vs. letter: *β* = −0.605, [−0.617, −0.594]).

### Individual analysis

**Figure 6:**
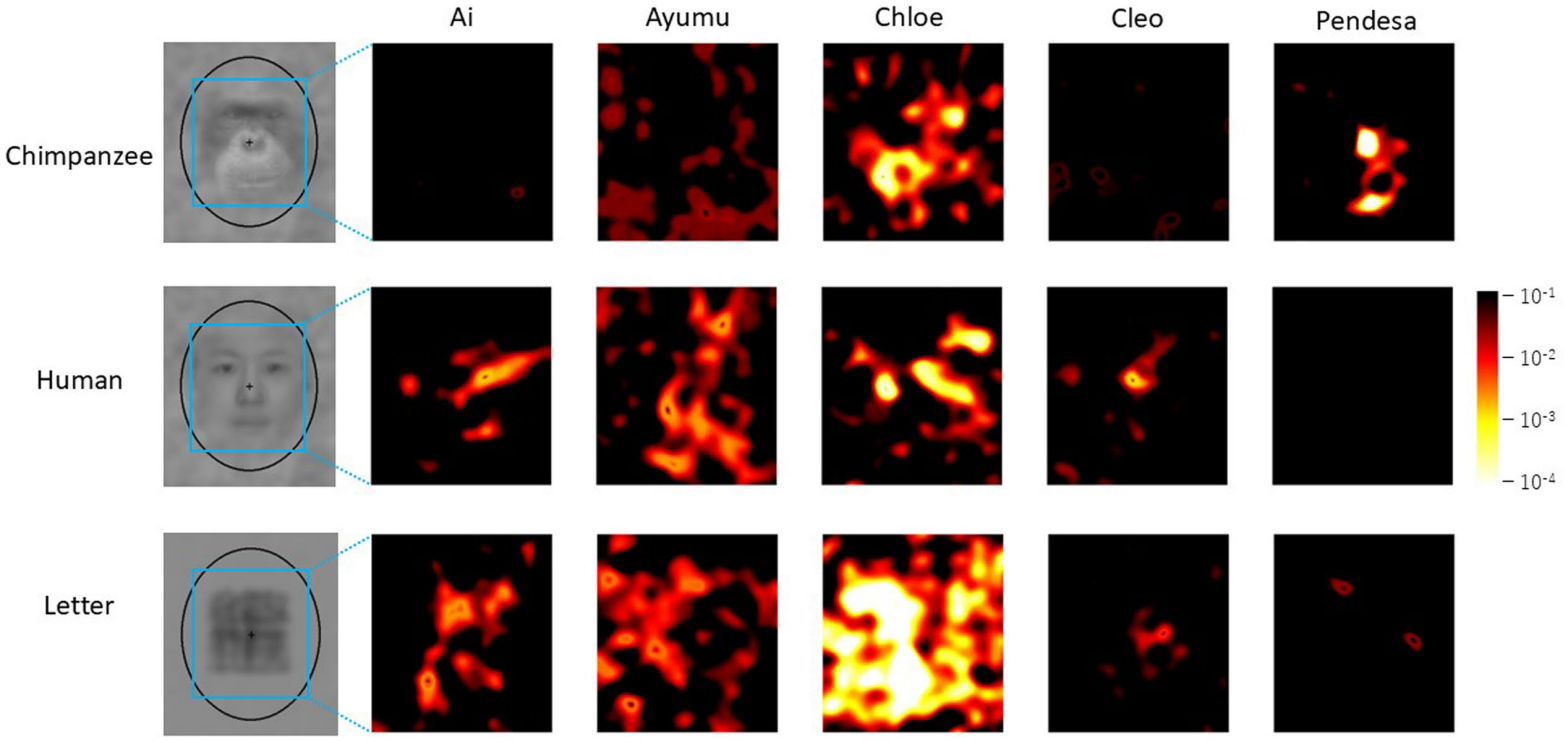
Difference image analysis based on data from each chimpanzee for each stimulus category. Only the heatmaps for each chimpanzee for each stimulus category are shown. Note that the color scale differs from the ones in Figures 4, 5.

Figure 6 presents the results of the difference image analysis for each individual. The *p*-value heatmaps, based on the actual selections of each individual, are displayed. Notable individual differences are observed in the heatmaps. For example, in the results for Ai and Cleo with chimpanzee faces and Pendesa with human faces, there are almost no significant regions in the heatmaps, similar to the heatmaps in Figure 5.

**Figure 7:**
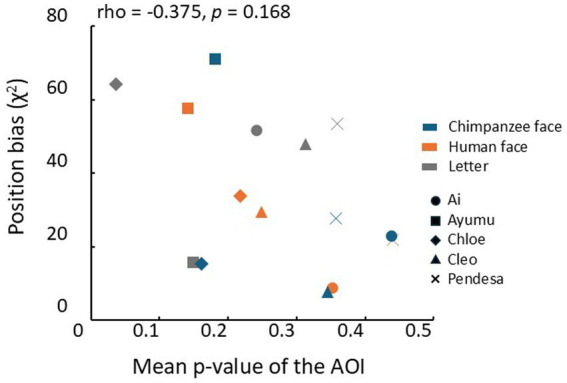
Relationship between the mean p-values in the AOIs and each chimpanzee’s position bias of choice response. Different symbols represent the results of different chimpanzees. Blue: chimpanzee face, orange: human face, gray: letters.rho: Spearman’s rank correlation coefficient.

One possible reason for these results is that the chimpanzees might have been guessing during the test trials. This possibility can be assessed by analyzing position biases as strong position biases might indicate guessing. Therefore, we performed the Spearman’s rank correlation analysis between the mean uncorrected *p*-values for each coordinate in the heatmaps and the position bias evaluated by chi-square values for each condition. The result is shown in Figure 7, but no significant correlation was found between the position bias and the mean *p*-values (Spearman’s rank correlation coefficient: rho = −0.375, *p* = 0.168, two-tailed).

### Comparison with the bottom-up face pareidolia experiment

The chimpanzees who participated in the present experiment had also taken part in a previous study by Tomonaga and Kawakami (2022). This earlier study used a visual search task similar to the one employed here to investigate bottom-up face pareidolia with face-like stimuli. In that study, they were required to detect a face-like object among photos of various non-face objects presented in a search display. The findings showed a significant difference in accuracy between unmanipulated upright stimuli and stimuli in which the top and bottom halves were horizontally misaligned, thereby disrupting the spatial configuration. Building on these findings, we explored the relationship between the accuracy difference for each individual in that study between upright and misaligned stimuli, and the mean *p*-value of the AOI for the two face conditions in the current top-down face pareidolia study. If the chimpanzees recognized the face-like objects as faces in the previous study, their performance would be expected to decline when the spatial configuration was disrupted, leading to a greater difference in accuracy compared to the intact stimuli. On the other hand, if they searched for faces in noise patterns in the present experiment, the mean *p*-value of the AOI would likely increase. Therefore, if the extent of face pareidolia exhibited by each chimpanzee influences both measures, a negative correlation between them would be expected.

**Figure 8:**
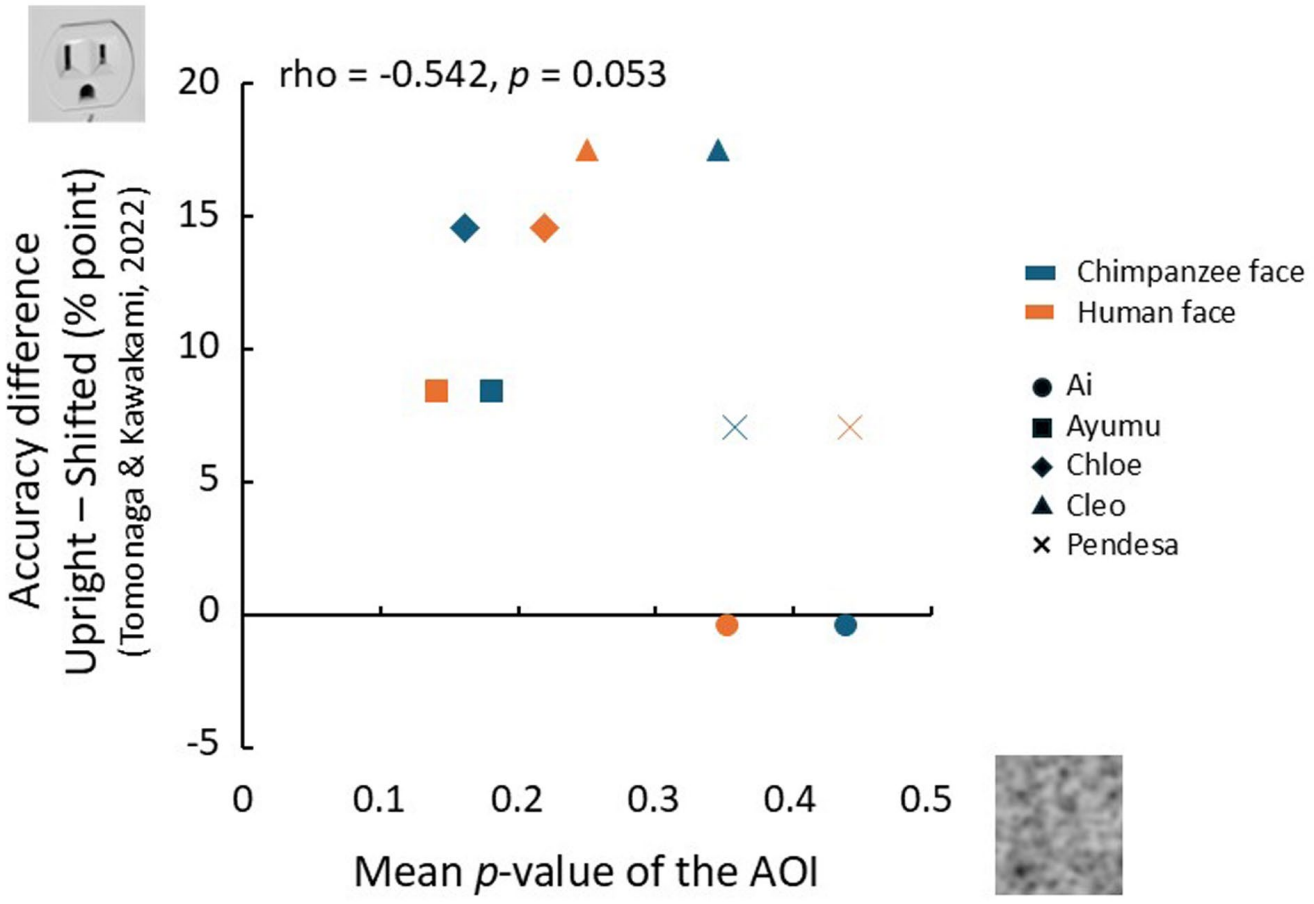
Rlationship between top-down face pareidolia (this experiment) and bottom-up face pareidolia (Tomonaga and Kawakami, 2022). The horizontal axis represents the mean p-values in the AOIs, and the vertical axis represents the differences in accuracy (percent points) between the upright and misaligned conditions for face-like stimuli.

The results of this analysis are presented in Figure 8. The vertical axis represents the difference in the percentage of correct trials from the study by Tomonaga and Kawakami (2022), while the horizontal axis represents the mean p-value of the AOI in the present experiment. Although the small sample size limits the strength of our conclusions, a near-significant correlation was observed between the bottom-up face pareidolia index and the top-down face pareidolia index (rho = −0.542, *p* = 0.053, one-tailed).

### Effect of response times

The response times in test trials were significantly longer than those in baseline trials, suggesting that the chimpanzees did not respond by guessing. However, previous studies have shown that in situations where a speed-accuracy trade-off does not occur, chimpanzees tend to exhibit longer response times for incorrect responses compared to correct ones (Tomonaga and Matsuzawa, 1992; Tomonaga et al., 2023). In other words, taking more time to respond does not necessarily mean that the chimpanzees were able to choose the “correct” stimulus. On the contrary, longer response times may indicate that they found nothing meaningful. This is also the case for humans. Hansen et al. (2010) excluded data with longer response times (e.g., those longer than the median) from their analyses. This data screening was based on research showing that object or face recognition generally occurs within a relatively short time frame (Johnson and Olshausen, 2003; Quiroga et al., 2005; Rekow et al., 2022; Wardle et al., 2020).

Following these findings, we further investigated the effects of response times on the current chimpanzee data. For each chimpanzee, the median reaction time for test trials in each stimulus condition was calculated (see symbols in Figure 3), and the data were divided into the two groups: those with response times equal to or shorter than the median (faster RTs), and those with response times longer than the median (slower RTs). The analyses of difference images were then conducted using GLMMs, both for the combined data of all chimpanzees and for each individual separately, as in the previous difference image analyses.

**Figure 9:**
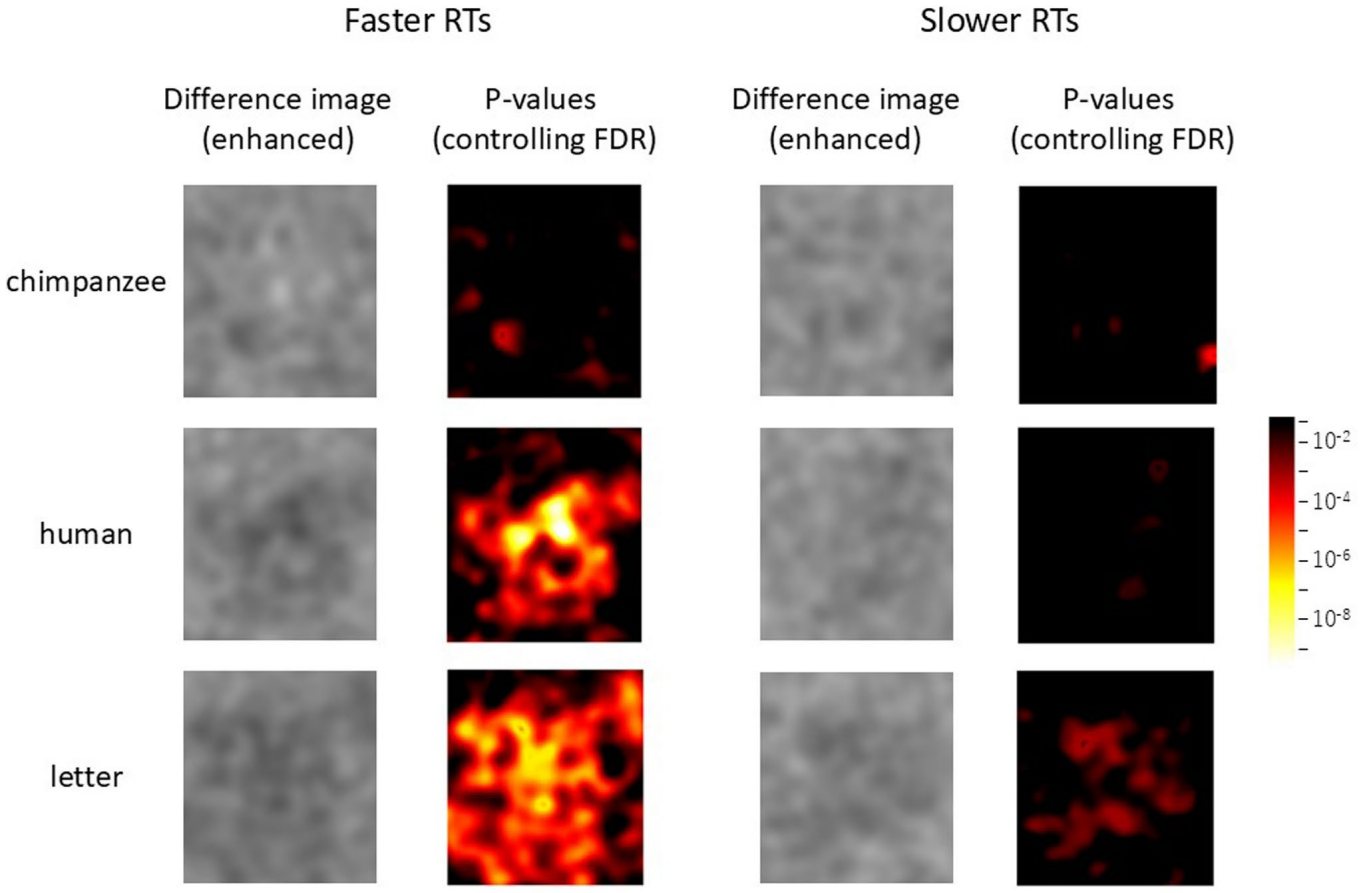
Effect of response times on face pareidolia. Difference image analysis based on the data from all chimpanzees. Left: faster response times, right: slower response times. The color scale is the same as Figures 4, 5.

**Figure 10:**
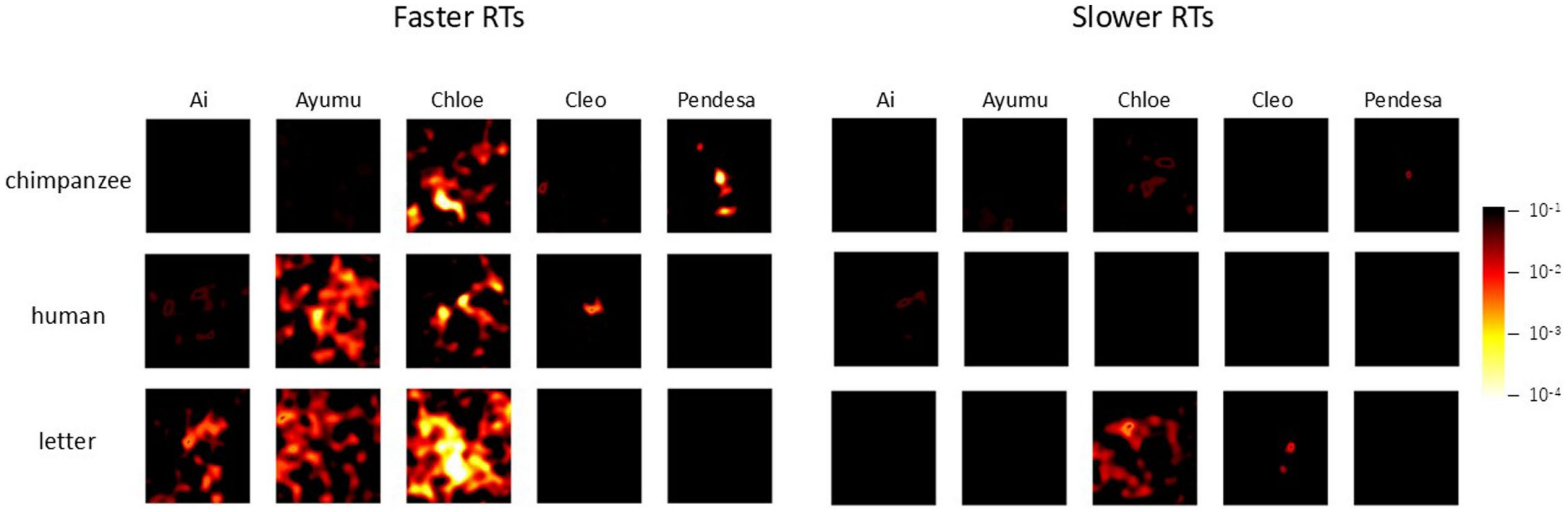
Effect of response times on face pareidolia. Difference image analysis based on each chimpanzee’s data. Left: faster response times, right: slower response times. The color scale is the same as Figure 6.

The results for all chimpanzees are shown in Figure 9. The left and right panels represent the results for faster RTs and slower RTs, respectively. In each panel, the left side displays the enhanced difference images cropped with the AOI, while the right side shows *p*-value heatmaps based on the GLMM results. As can be seen from the figure, clearer structures were observed in the difference images obtained from the shorter RT data across all stimulus conditions. Figure 10 presents the results of the analysis conducted for each individual, where similar findings were obtained.

## Discussion

In the present experiment, after training the chimpanzees to repeatedly detect stimuli from a specific category in the oddity discrimination task, they were required to select one pattern from three noise patterns during the test sessions. As a result, statistically significant structures were observed in the difference images between the averaged patterns they selected and those they did not select. In contrast, when similar analyses were conducted using patterns randomly selected by a computer, no such structures were observed.

Response times in the test trials were significantly longer than those in the baseline trials, suggesting that the chimpanzees were unlikely to have selected the noise patterns by guessing. Although individual differences were observed in the difference images for each category, no correlation was found between the clarity of the structures (assessed by the mean *p*-values across the entire AOI) and the response biases in each individual. These results suggest that even when no clear structures were detected, the chimpanzees did not select the patterns by guessing.

However, further analysis revealed that longer response times did not necessarily mean that the chimpanzees were able to find something meaningful in noise patterns. When the data for each chimpanzee were divided based on the median response times and analyzed, clearer structures were observed in the difference images for shorter response times compared to longer ones. This finding suggests that while longer response times may indicate deeper decision-making processes, they do not necessarily lead to improved detection accuracy. In other words, the current data suggest that there were trials where the chimpanzees were unable to detect faces or letters. Response times may indirectly indicate the certainty of the decisions made by the chimpanzees in each trial (Tomonaga et al., 2023; Tomonaga and Matsuzawa, 2002).

Conversely, it may be possible to further filter the data using such a certainty indicator of accuracy made by observers. Many studies have reported that non-human animals are capable of monitoring the accuracy of their own choices (Smith et al., 1995, 1997; see also Fujita, 2010; Hampton, 2009; Tomonaga et al., 2023 for review). For example, in a separate study, the chimpanzees who participated in the present experiment spontaneously displayed a behavior where, immediately after making a response, they would look back at the food dispenser to check whether it was operating correctly. This looking-back behavior occurs even in the absence of auditory feedback, such as the sound of the dispenser or a chime for correct responses. Moreover, it has been found that this behavior occurs more frequently when their response was correct than incorrect (Tomonaga et al., 2023). Thus, this looking-back behavior could be used as a spontaneous indicator of the accuracy. Unfortunately, in the present experiment, the chimpanzees’ behavior during the trials was not recorded, so filtering the data based on this behavior was not possible. However, future studies could incorporate procedures that allow for the use of such behaviors as part of the analysis.

Regardless, the present results strongly suggest that the chimpanzees were attempting to find “something” within the noise patterns that did not contain embedded stimuli from any category. When examining the *p*-value heatmaps and the extracted patterns, a top-heavy structure was observed in the human-face condition. However, the positions of the components were slightly misaligned compared to the positions of the eyes and mouth in the averaged images. In contrast, for the chimpanzee-face condition, the significant areas were smaller than those for the other categories. Nevertheless, the highlighted area corresponded to the bright regions around the chimpanzee’s mouth. For the letter category, significant areas were concentrated in the center of the image, which is similar to results observed in humans (Liu et al., 2014; Rieth et al., 2011).

Liu et al. (2014) conducted a correlation analysis based on the results of a one-dimensional Fourier analysis of each image to evaluate the similarity between the structures observed in the difference images and the average images of each category. Their analysis found significant correlations only between the average and difference images of faces and between those of letters, suggesting a similarity between the average and difference images. We also conducted similar correlation analyses to those by Liu et al. (2014) and additional analyses using Euclidean distances between difference images on the results obtained from chimpanzees, but unfortunately, no systematic trends were observed.

The key difference between the present experiment and the study by Liu et al. (2014) lies in the control of the observer’s gaze. In the human experiment, only one image was shown at a time with a consistent fixation point, which allowed some control over the observer’s gaze. If the fixation point had effectively controlled the observer’s gaze, the one-to-one correspondence among the selected images at each pixel would likely have been maintained. In contrast, in our chimpanzee experiment, three stimuli were presented in random positions on each trial, without any control over gaze. In such a free-viewing condition, the chimpanzees may have searched for faces by focusing on different regions of each image in each trial. Although an elliptical frame and a central point were added to each noise pattern in the present experiment to somewhat control spatial attention, this may have been insufficient. The slight shifts in the fixation point across trials may have blurred the average image and, as a result, diminished the clarity of the structure in the difference images.

Another possibility is that the effects of repetition or sequential priming on “expectancy” may not have been as strong in controlling the chimpanzees’ behavior as the top-down control provided by explicit verbal instructions in humans. As mentioned in the introduction, because it is impossible to give verbal instructions, a standard method for enhancing top-down control, to non-human animals like chimpanzees, we attempted to enhance expectancy through repetitive presentations, a bottom-up approach. The chimpanzees likely made their choices based on some expectancy, as evidenced by their prolonged response times during the test trials. However, it cannot be ruled out that this bottom-up priming of behavior may lead to fundamentally different outcomes compared to top-down control. This might be also the case for humans. Takahashi and Watanabe (2015) reported a contrary finding, showing that top-down control of face pareidolia is insufficient with verbal instruction alone, and that baseline trials with face stimuli, as in our experiment, are necessary.

To further explore this issue, I propose two approaches. The first is to use a matching-to-sample task, which aligns more closely with top-down control. In this task, a sample stimulus identical or categorically similar to the correct choice is presented before the alternatives. This sample may function similarly to verbal instructions in human experiments, potentially enabling top-down control over chimpanzees’ face perception by presenting it before the choice stimuli in test trials.

The second approach involves using humans as a positive control for procedures used (or to be used) in chimpanzees (cf. Tomonaga and Imura, 2023a, 2023b). The repetitive presentations used in this study, as well as the proposed matching-to-sample method, may not effectively enable top-down control of face perception regardless of the participant species. To rule out this possibility, positive control experiments with humans are necessary. Specifically, if clear structures are observed in the difference images under these tasks in humans, it would indicate that these tasks are effective at the procedural level for examining top-down control of face perception. Further investigation of these approaches is warranted.

Lastly, it is important to acknowledge the small sample size in the present experiment. While the number of trials per individual in a study with humans by Liu et al. (2014) was comparable to that in our experiment, their study included significantly more participants. This discrepancy may have influenced the robustness of the present findings. This concern is further highlighted by the relatively large individual differences observed in our experiment, as shown in Figures 6, 10. Although we employed GLMMs to account for individual differences, future studies with a larger sample size will be necessary to replicate and strengthen these results.

In the present experiment, five chimpanzees participated, which was due to facility constraints and the difficulty of the discrimination task. In other studies conducted in our laboratory, the number of participants has never exceeded 10 (Gao et al., 2020; Gao and Tomonaga, 2018; Gao and Tomonaga, 2020a; Gao and Tomonaga, 2020b; Kawaguchi et al., 2020; Tomonaga and Kawakami, 2022; Ludwig et al., 2011; Tomonaga et al., 2024; Xu et al., 2024; Yokoyama et al., 2024). Similarly, in comparative cognitive research conducted at other laboratories, studies involving great apes typically include fewer than 10 individuals per species (Chertoff et al., 2018; Kret et al., 2016; Krupenye et al., 2016; Parr et al., 1998; Parr and Waller, 2006). Studies with more than 10 participants are rare and often involve tasks that do not require complex learning skills, such as eye-tracking (Kano and Call, 2014). To overcome the issue of sample size, multi-facility collaborations will likely be essential in the future (ManyPrimates, 2019a, b).

Despite the individual differences observed, a weak but noticeable correlation was found between those in the top-down and bottom-up face pareidolia experiments. The chimpanzees in the present study exhibited clear inversion and misalignment effects when using facial stimuli (Dahl et al., 2013; Tomonaga and Kawakami, 2022), suggesting that individual differences in face recognition itself are unlikely. Additionally, no clear relationship was observed between the indices from both experiments and the ages of the participants. Further research with a larger sample size will be required to better explore the relationship between bottom-up and top-down face pareidolia.

It may also be possible to retain the current sample size while significantly increasing the number of trials per individual, similar to previous classification-image studies (e.g., Gosselin and Schyns, 2003; Hansen et al., 2010). However, this approach carries the potential risk that, with prolonged exposure to test sessions, the chimpanzees may learn that no stimulus is embedded in the test trials, resulting in random guessing. This possibility warrants careful consideration in future experimental designs.

In conclusion, the present results suggest that the chimpanzees tried to detect something meaningful in noise patterns when repeatedly exposed to a specific stimulus category. These results may correspond to top-down pareidolia observed in humans. While it is likely that what they detected shares features with the repeatedly presented stimulus category, further methodological improvements and studies with larger sample sizes will be necessary to draw firmer conclusions.

## Data Availability

The datasets presented in this study can be found in online repositories. This data can be found at Figshare: https://doi.org/10.6084/m9.figshare.27191841.v1.

## Footnotes

^1^ https://shigen.nig.ac.jp/gain/
